# Cardiovascular magnetic resonance at 7.0 Tesla in patients with hypertrophic cardiomyopathy - a pilot study

**DOI:** 10.1186/1532-429X-17-S1-Q108

**Published:** 2015-02-03

**Authors:** Marcel Prothmann, Etham Shahid, Agnieszka Toepper, Florian von Knobelsdorff-Brenkenhoff, Andreas Graessl, Jan Rieger, Darius Georg Lysiak, Christof Thalhammer, Till Huelnhagen, Thoralf Niendorf, Jeanette Schulz-Menger

**Affiliations:** 1 Cardiology, Charité. Medical Faculty of Humboldt-University Berlin ECRC and HELIOS Clinics, Berlin, Germany; 2 Berlin Ultrahigh Field Facility (B.U.F.F.), Max-Delbrueck Center for Molecular Medicine, Berlin, Germany; 3 MRI.TOOLS GmbH, Berlin, Germany

## Background

Cardiovascular Magnetic Resonance (CMR) is known to offer additional morphologic information in Hypertrophic Cardiomyopathy (HCM). CMR based myocardial tissue differentiation and the detection of small morphological details is of proven clinical value. MR at a magnetic field strength of 7 Tesla holds the promise to enhance spatial resolution and anatomical detail, but is currently primarily used in brain imaging [1]. CMR at 7.0T has been applied in healthy volunteers [2,3]. Here we examine the feasibility of CMR at 7T in patients and demonstrate its capability for the visualization of subtle morphological details which are elusive in today's clinical CMR practice.

## Methods

We screened 131 patients with HCM. 13 Patients with HCM (9 male, 56 ±31 years) and 13 healthy subjects (9males, 55±31years) were scanned at 7T and 3T, a subgroup at 1.5 T (Siemens, Erlangen, Germany). Cine imaging for the assessment of function and morphology was performed at all field strengths (voxel size 1.4x1.4mm^2^ with slice thickness at 7T: 2,5mm and 4mm; at 3 and 1.5 Tesla with 6mm). Late gadolinium enhancement (LGE) was performed at 3T for imaging of fibrosis. Fat-water imaging techniques were employed at 1.5 T for fat analysis.

## Results

All scans were successful and evaluable. At 3T LV quantification were similar in short axis view and biplane approach (LVEDV, LVESV, LVMASS, LVEF) (p=0.232; p=0.437; p=0.549; p=0.118). There was no significant difference between 3T and 7 T (p=0.111; p=0.167; p=0.119; p=0.204). Typical LGE was detected in 12/13 (92%) HCM patients. High spatial resolution CINE imaging at 7.0 T revealed deep but small hyperintense myocardial crypts in the regions for which LGE was detected at 3T. Myocardial crypts occurred in 7/13(54%) of HCM patients. The presence of fat was excluded as a cause for the hyperintense signal. No crypts were detected for 2D CINE imaging at 3T.

## Conclusions

7 Tesla MRI is feasible in patients with HCM. High spatial resolution CINE imaging at 7.0 T affords the detection of subtle myocardial crypts in regions of extended hypertrophy and LGE.

## Funding

N/A.

**Figure 1:**
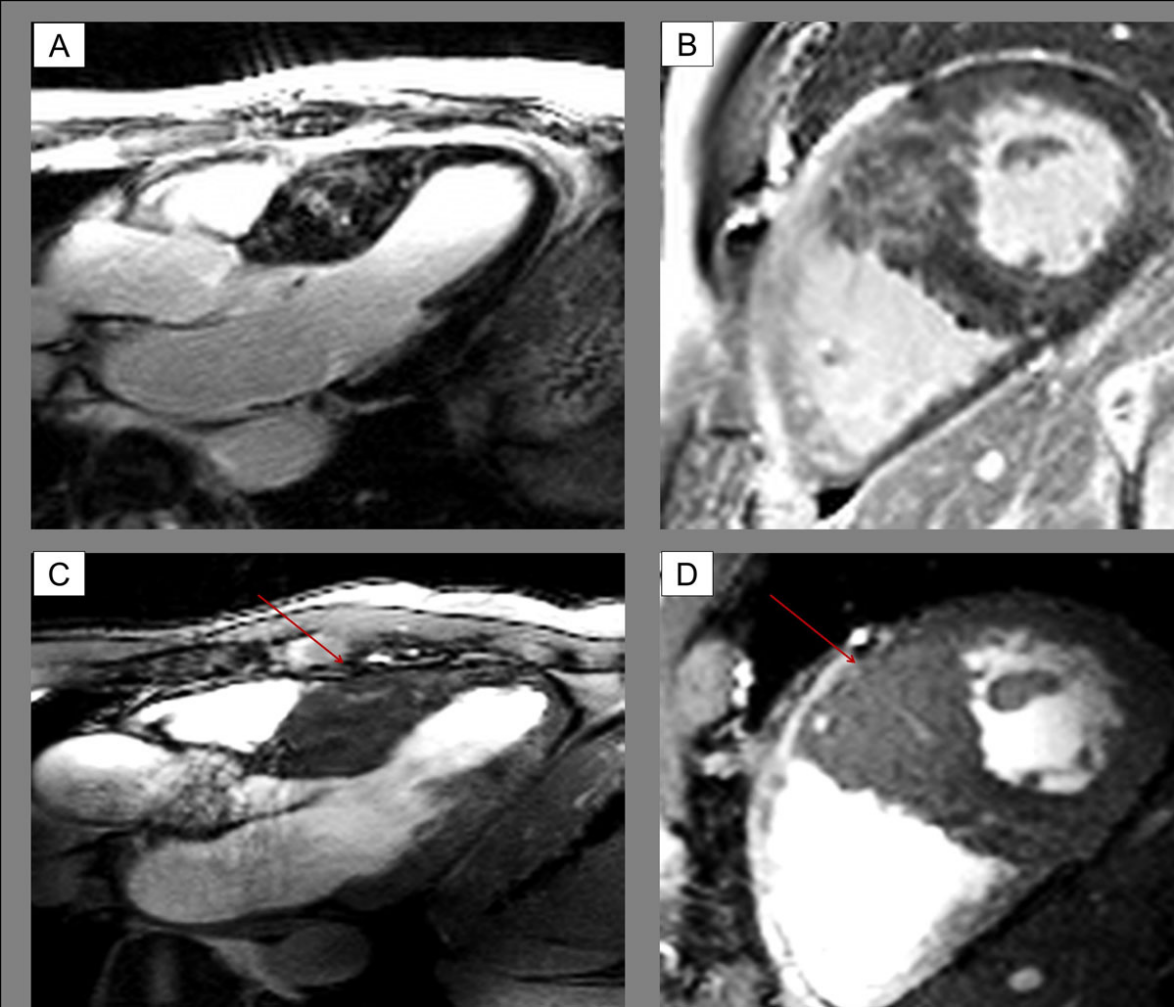
Case example with myocardial crypts- hyperintense signal displays at LGE-images is partly explained by blood. A-B: LGE-imaging at 3T. C-D: CINE GRE at 7T

**Table 1:** Comparison of LV-function at different field strengths

Mean	3T (biplane)	7T (biplane)	p-value
LVEDV (ml)	139.9	136.7	0.232

LVESV (ml)	53.0	51.2	0.437

LVM (g)	187.0	174.9	0.549

LVEF (%)	62.9	59.9	0.118

## References

[B1] Sinnecker T Distinct lesion morphology at 7-T MRI differentiates neuromyelitis optica from multiple sclerosis. Neurology 2012 79 7 708 14 10.1212/WNL.0b013e3182648bc8 22855861

[B2] von Knobelsdorff-Brenkenhoff F Cardiac chamber quantification using magnetic resonance imaging at 7 Tesla--a pilot study Eur Radiol 2010 20 12 2844 52 10.1007/s00330-010-1888-2 20640427

[B3] von Knobelsdorff-Brenkenhoff F Assessment of the right ventricle with cardiovascular magnetic resonance at 7 Tesla J Cardiovasc Magn Reson 2013 15 23 10.1186/1532-429X-15-23 23497030 PMC3621368

